# On-Line Real-Time Monitoring of a Rapid Enzymatic Oil Degumming Process: A Feasibility Study Using Free-Run Near-Infrared Spectroscopy

**DOI:** 10.3390/foods10102368

**Published:** 2021-10-05

**Authors:** Jakob Forsberg, Per Munk Nielsen, Søren Balling Engelsen, Klavs Martin Sørensen

**Affiliations:** 1 Food Analytics and Biotechnology, Department of Food Science, University of Copenhagen, Rolighedsvej 26, DK-1958 Frederiksberg C, Denmark; se@food.ku.dk (S.B.E.); kms@food.ku.dk (K.M.S.); 2 Novozymes, Oils & Fats Application Research, Biologiens Vej 2, DK-2800 Kongens Lyngby, Denmark; pmn@novozymes.com

**Keywords:** Near-infrared spectroscopy, process analytical technology (PAT), process control, processing technology, chemometrics, vegetable oil, oil refinement, variable selection

## Abstract

Enzymatic degumming is a well established process in vegetable oil refinement, resulting in higher oil yield and a more stable downstream processing compared to traditional degumming methods using acid and water. During the reaction, phospholipids in the oil are hydrolyzed to free fatty acids and lyso-phospholipids. The process is typically monitored by off-line laboratory measurements of the free fatty acid content in the oil, and there is a demand for an automated on-line monitoring strategy to increase both yield and understanding of the process dynamics. This paper investigates the option of using Near-Infrared spectroscopy (NIRS) to monitor the enzymatic degumming reaction. A new method for balancing spectral noise and keeping the chemical information in the spectra obtained from a rapid changing chemical process is suggested. The effect of a varying measurement averaging window width (0 to 300 s), preprocessing method and variable selection algorithm is evaluated, aiming to obtain the most accurate and robust calibration model for prediction of the free fatty acid content (% (*w*/*w*)). The optimal Partial Least Squares (PLS) model includes eight wavelength variables, as found by rPLS (recursive PLS) calibration, and yields an RMSECV (Root Mean Square Error of Cross Validation) of 0.05% (*w*/*w*) free fatty acid using five latent variables.

## 1. Introduction

**Figure 1:**
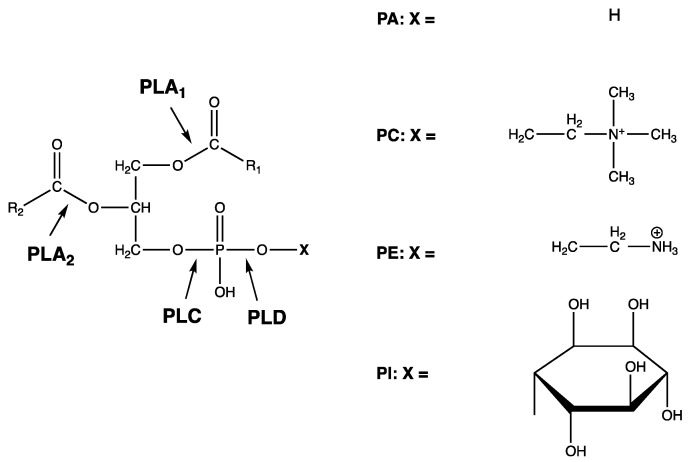
Chemical overview of the action of different types of phospholipases (PL), including PLA_1_, PLA_2_, PLC and PLD. R_1_, R_2_: Fatty acid residues. PA = phosphatidic acid, PC = phosphatidylcholine, PE = phosphatidylethanolamine, PI = phosphatidylinositol.

With an increasing demand for vegetable oils, both in the food industry and for the production of biofuels, there is a call for an effective and environmentally friendly method to remove naturally occurring phospholipids, known as “gums”, during oil refinement [1]. Degumming has traditionally been carried out using water followed by a chemical refining step, such as acid degumming, but is today generally conducted using enzymatic methods, which offer both higher oil yield and lower environmental impact [2]. Degumming is an essential step in the refinement process of vegetable oils, as it serves to reduce the content of phospholipids. Enzymatic degumming is well established, and different types of phospholipases are commercially available. Figure 1 shows an overview of the action of different types of phospholipases. The use of phospholipase type A (PLA) is an efficient way to reduce the content of phospholipids by hydrolyzation into lyso-phospholipids and free fatty acids (FFA). This enzymatic action results in higher oil yield, both due to higher FFA content in the oil and due to less entrapment of glycerides in the gum phase [2,3,4].

In industry, enzymatic degumming is typically carried out in continuous stirred compartmentalized tank reactors with a holding time of 4–6 h depending on the oil origin and initial phospholipid content. Typically, the process is run according to a recipe with a fixed holding time and monitored by taking out grab samples for laboratory analysis [2]. The industry standard for analysis is determination of FFA content by titration [5], which is time-consuming, labor intensive and requires use of chemicals. In addition, the reproducibility of the method is questionable [5].

Near-infrared spectroscopy (NIRS) is used in multiple production processes to monitor different quality attributes in the vegetable oil processing industry. These include dry matter and titratable acidity [6], yield [7] triglycerides [8] and free fatty acid composition [9]. NIRS has several advantages in comparison to the chemical method: samples can be measured in few seconds, it is non-destructive and no sample preparation or pre-treatment is required. NIRS is an interesting candidate method for real-time monitoring of enzymatic degumming, and has previously been used to monitor FFA in biodiesel production [10,11] and to monitor transesterification reactions [12,13,14]. NIRS is especially advantageous due to its high speed and adaptability to on-line monitoring [15]. In addition, the combination of NIRS and multivariate analysis enables monitoring and control of several quality attributes from only one spectroscopic sensor.

Replacement of the current grab sample strategy with real-time NIRS monitoring during the enzymatic degumming process will greatly facilitate automatic optimization of the process. Acquisition of NIR spectra from a bioreactor can be either on-line or in-line, and depending on the process equipment and the type of process, each mode of measurement has its own advantages [15]. Traditional in-line fiber optic immersion probes may have limited suitability for the viscous oil matrix involved in the enzymatic degumming process. Hence, the on-line solution proposed in this study consists of a closed bypass loop with a flow cell, where the process flow goes through and returns to the reactor, giving several benefits in comparison to a fiber optic probe mounted directly in the reactor. An on-line bypass loop is very beneficial in mitigating formation of air in the reactor due to high stirring rates and allows for moving the viscous matrix through a sampling window. On-line NIRS enables the control of enzyme dosage and holding time by feedback PAT (Process Analytical Technology) control [16], ensuring that the process is always in its optimal state, which is beneficial for economic, production time/capacity and environmental/resource expenditure reasons. The holding time and required amount of enzyme is highly dependent on the state of the raw material, i.e., the initial phosphorus content of the oil, the enzymatic performance in the given environment and the current state of the process [2]. NIRS can facilitate this real-time and on-line monitoring of the current reaction state, leading to full control and optimization of the process.

Modern NIR instrumentation enables very fast acquisition of spectral scans during on-line monitoring. For interferometer-based instruments, where a spectrum is obtained by Fourier Transformation (FT) of the measured interferogram, a scan can typically be obtained in a few seconds. If several scans are averaged to reduce the measurement noise, the spectral noise can be approximated as $1/\sqrt{N}$, where *N* is the number of scans. Thus, to reduce the noise by 50%, the number of scans must be increased fourfold. The signal to noise ratio (SNR), or sensitivity, can be approximated by $\sqrt{N}$. However, SNR only improves until the point where instrumental drift or other minor factors will start to dominate the random noise [17,18]. In addition, the duration of measurement will at some point start to influence the apparent process dynamics measured. In practice, measurement duration will represent a compromise between process dynamics and measurement uncertainty [16]. To achieve an acceptable level of noise (measurement uncertainty) during NIRS analysis, it is common to collect and average a reasonable number of scans, causing a single measurement campaign to last several minutes. In case of a fast-changing chemical process, i.e., a process with a high reaction rate such as industrial enzymatic degumming, the average of these scans may lead to misinterpretation of the current state of the process, since the process may have changed considerably during acquisition from the first to last scan. The resulting average spectra simply does not represent the process state as it was at the timepoint of the last scan but rather the “chemical average” of the process kinetics as it happened during the whole data acquisition period. This difference between the final produced averaged spectra and the change in process presents an obvious problem when trying to regulate the process to an optimal setpoint. This paper aims at investigating the effect of the compromise between process dynamics and measurement uncertainty by varying the spectral averaging strategy to be adapted to the process dynamics.

As an alternative to acquiring NIRS measurements by averaging a fixed number of scans, a method where the instrument is set to generate scans in a free-run mode is proposed. In such a setup, there is no traditional beginning or end of a measurement, but just an endless stream of individual spectral scans. By applying a window function in the time-domain on this stream, it is possible to control how spectra are collated (averaged) prior to subsequent data analysis. By proper weighing of the prior scans in the window, not only will spectral noise (measurement uncertainty) be reduced as described above, but the changing reaction kinetics of the process can be considered as well. The way prior samples are weighted in the window is controlled by the width of the window. In the present study, we will examine the effect of varying the window width in the range of 0 to 300 s in intervals of 30 s.

In addition, the effect and performance of each window width based on PLS calibration models of FFA content is evaluated. This includes evaluation of different spectral preprocessing techniques and application of two different variable selection algorithms to obtain the most robust calibration model. The model performance is evaluated based on RMSECV (Root Mean Square Error of Cross Validation) and number of latent variables included and is illustrated using response surface plots.

## 2. Materials and Methods

Two batches of crude oil originating from the United States were used in the experiment. The free fatty acid content was determined at arrival at the laboratory by a slightly modified version of the AOCS Ca 5a-40 official method [5] working with a smaller sample volume (1.0 g). Oil A was a soybean oil with a FFA content of 0.57%. Oil B was a rapeseed oil with a FFA content of 1.23%. The oils were stored at 5 °C until onset of the experiment. The phospholipase A1 type enzyme (Quara^®^ LowP, Novozymes A/S, Denmark) was used as the reaction enzyme. All other chemicals and reagents used in this study were purchased from Sigma Aldrich (Düsselforf, Germany).

### 2.1. Enzymatic Degumming

The two batches of crude oil were enzymatically degummed using a laboratory scale batch system consisting of a 250 mL blue cap flask as the reactor and a temperature-controlled water bath with magnetic stirring (Model T100, Grant Instruments Ltd., Cambridge, UK).

A total of 200 g of crude oil was prepared in the blue cap flask and heated to 70 °C at 500 rpm magnetic stirring in a water bath. The heated oil was treated with 650 ppm of citric acid solution (50% *w*/*w*, citric acid reagent grade ≥ 99.5%) and mixed in an ultrasonic bath for 5 min at 60 °C (Branson 3510 Ultrasonic Cleaner, Marshall Scientific, Hampton, NH, USA). Upon 15 min of incubation at 70 °C, 3% of Milli-Q^®^ water was added, followed by the addition of 30 ppm of the phospholipase A1 enzyme Quara^®^ LowP. The mixture was ultrasonicated for another 5 min at 60 °C, and the reactor was placed in the water bath at 70 °C for reaction. The reaction was monitored for three hours.

### 2.2. NIRS Measurements

**Figure 2:**
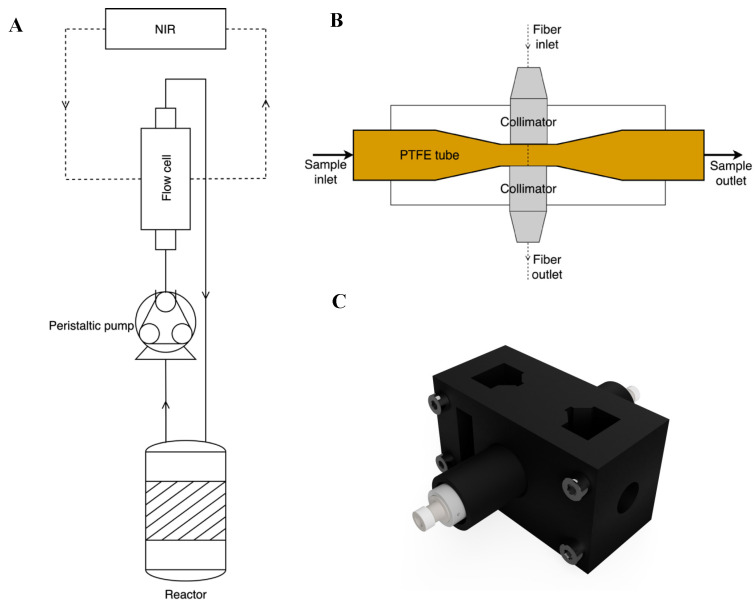
(**A**) Layout of sample loop. The experimental setup includes a stirred reactor, a peristaltic pump, a flow cell and a NIRS instrument. (**B**) Cross-sectional layout of flow cell. (**C**) Assembled flow cell with collimators.

A sample loop consisting of a flow cell and a peristaltic pump (Minipuls 3, Gilson Inc, Middleton, WI, USA) as shown in Figure 2A was used. During degumming, the oil was pumped at 48 rpm in silicone tubing (I.D. 3.18 mm), through the flow cell, equal to a flow rate of approximately 28 mL/min.

Spectroscopic data was collected with a Quant FT-NIR (ABB MB3600 FT-NIR) Fourier Transform spectrometer with an InGaAs detector (Q-interline, Tølløse, Denmark) using the HORIZON MB Pro software (ABB, Zurich, Switzerland). A 3D-printed flow cell equipped with a Teflon (PTFE) 7 mm OD/6 mm ID tube was used as sample cell. Teflon was chosen as it is transparent to near-infrared radiation and has excellent performance with the viscous matrix. The light path length was fixed at 2 mm. The flow cell was equipped with two collimators (model F240SMA-1550, Thorlabs, Newton, NJ, USA) connected to the instrument by 3 m low-OH process grade fiber optical cables (Thorlabs, Newton, NJ, USA). A graphical representation of the flow cell is shown in Figure 2B,C.

The spectrometer was set to record scans in the range from 11,000 cm^−1^ to 3900 cm^−1^ (≈910–2775 nm), with a resolution of 8 cm^−1^ and a digitalization of 4 cm^−1^, resulting in 1584 data points per scan. A background spectrum of the empty Teflon tube was obtained before the enzymatic degumming process was started.

The software was set to obtain one spectrum as frequent as possible, resulting in a scan every 2–8 s due to software limitations, resulting in collection overhead. In total 3979 and 3913 spectra were obtained from batch A and B, respectively.

### 2.3. Reference Sampling

Reference samples were grab-sampled every fifth minute in the first hour and every tenth minute thereafter throughout the experiment. Approximately 2 mL was transferred directly from the sample loop silicone tube to a preheated Eppendorf tube. The enzyme was inactivated by heating at 99 °C for a minimum of 30 min using an Eppendorf Thermomixer (Eppendorf, Hamburg, Germany) immediately after sampling. The FFA content was determined using a slightly modified version of the AOCS Ca 5a-40 official method [5] working with a sample volume of 1.0 g. The volume of alkali used for titration to neutralize the acidity in the samples was used to calculate FFA expressed as oleic acid using the formula in the official method. The relative standard deviation (RSD) of the titration method was 14.57% in the range of 0.1–1% FFA and 9.84% in the range of 1.0–2.0% FFA [5]. In total, 25 reference samples were obtained from each batch and were stored at 5 °C for 5–7 days until analysis.

### 2.4. ^31^P-NMR Analysis

The crude oils were analyzed using ^31^P-NMR to quantify the different phosphor-containing species present. An oil sample of approx. 250 mg was transferred to a 2 mL Eppendorf tube. An aliquot of 500 µL 2 mg/mL TPP (triphenyl phosphate) in MeOH was added as internal standard, together with 500 µL of chloroform (CDCl_3_) and 500 µL 0.2 M Cs-EDTA buffer. The tube was shaken for 30 s and centrifuged at 13,400 rpm for 1 min to obtain phase separation. An aliquot of 700 µL of the organic phase was transferred to a 5 mm NMR tube for analysis. All ^31^P-NMR spectra were acquired at 298 K on a Bruker Avance IIIHD 400 MHz spectrometer equipped with a BBFO room temperature probe. Each spectrum was acquired using 128 scans with a relaxation delay of 5 s. The concentration of each species was calculated as ppm P, i.e., mg elemental P per kg oil sample.

### 2.5. Data Analysis and Model Calibration

The data analysis was performed in MATLAB R2021a software (The Mathworks, Inc., Natick, MA, USA) using the PLS_Toolbox version 8.9.1 (Eigenvector Research Inc., Wenatchee, WA, USA).

### 2.6. Data Screening and Removal of Spectral Outliers

Visual inspection of the spectra revealed that several of the acquired spectra were noisy due to particles and bubbles passing through the experimental flow cell. These scans were considered outliers and removed programmatically if (1) the absorbance was higher than 2.99 for more than eight spectral wavelengths (indicating a blockage) and/or (2) a scans’ standard deviation of absorbance evaluated over all wavelengths was found to have a standard deviation lower than 0.15 (air bubble or no sample present).

Data was subsequently averaged in the process time domain by application of a moving arithmetic average algorithm with a varying window width. The window width varied from 0 to 300 s in 30 s interval, always operating on prior data. The moving average algorithm applied the same weight to all spectra in the window.

### 2.7. Preprocessing of the Spectra

In order to reduce the amount of optical scatter in the acquired scans, three preprocessing methods were examined to determine if any significant reduction in predictive error could be obtained [19]: MSC (Multiplicative Scatter Correction) [20], EMSC (Extended Multiplicative Scatter Correction) [21] and Savitzky-Golay (SG) second order smoothing and second derivative, with widths of 15, 31 and 59, respectively [22]. All data were mean centered (MC) after preprocessing, and the three preprocessing methods were compared to raw mean centered spectra. The spectral range of 1100–2200 nm was used for model calibration.

### 2.8. Principal Component Analysis (PCA)

Data were explored by Principal Component Analysis (PCA) to evaluate the process kinetics and changes over time and to retrieve information about systematic variation in the dataset [23]. A total of 66 PCA models were calculated, representing each combination of window width (0 to 300 s with 30 s intervals) and preprocessing (MC, SG15,2,2 + MC, SG31,2,2 + MC, SG59,2,2 + MC, MSC + MC, EMSC + MC).

### 2.9. Partial Least Squares (PLS) Regression

Partial Least Squares (PLS) regression [24] was performed to evaluate the relationship between the near-infrared spectra and the FFA reference values obtained from titration. The preprocessed free run data were reduced to align with the response variables using time stamps, and all non-aligned spectra were excluded. The two batches were combined into one dataset of size 48 spectra × 1178 variables. One PCA model for each preprocessing method was calculated, and outliers were identified programmatically based on the confidence of scores in LV1 (latent variable) and LV2, explaining on average 93% of the data across all window widths and preprocessing methods. Samples outside the 95% (± 2 standard deviations) confidence were removed, and finally the intersection of outliers across the six different preprocessing methods was removed to obtain datasets of equal size for comparison. The final dataset for PLS regression included 45 spectra of 1178 variables.

Calibration models were cross validated using a segmented venetian blinds cross validation, with six segments of four samples each, equivalent to 15–30 min reaction time. The calibration models were evaluated based on the number of components included, the RMSECV (Root Mean Square Error of Cross Validation) and the correlation (as coefficient of determination, r^2^). The number of latent variables to include was initially estimated by evaluation of the data and then fixed for easier comparison between combinations of preprocessing and average window width.

### 2.10. Variable Selection

Variable selection was carried out by both recursive PLS (rPLS) [25] and interval PLS (iPLS) [26]. Recursive PLS uses the regression vector as a base for weighting the individual variables, taking advantage of the fact that the regression vector reflects the importance of the variables [25]. In this study, rPLS models including one to five latent variables were calculated for each combination of window width and preprocessing, and the included variables resulting in the lowest RMSECV were chosen for modelling. Interval PLS performs selection of variable intervals based on the RMSECV value obtained for each individual interval of variables and sequentially selects the intervals resulting in the lowest RMSECV [26]. In this study, the wavelength region (1100–2200 nm) was divided into segments of 100 variables using forward selection.

## 3. Results

### 3.1. Statistics of Reference FFA Measurements

Basic statistics of the reference values obtained by FFA analysis are shown in Table 1. Two batches were monitored during degumming, using soybean and rapeseed oil as the raw material. Rapeseed oil consists mainly of mono-unsaturated fatty acids, whereas soybean oil has a higher content of saturated and poly-unsaturated fatty acids [27]. The initial FFA content was significantly higher in the rapeseed oil than in the soybean oil, and both levels were in accordance with average oil compositions [28]. For regression modelling, the data from the two batches were combined into one dataset, resulting in a relative standard deviation of 30.3%, which is judged to be sufficient to establish feasible calibration models.

**Figure 3:**
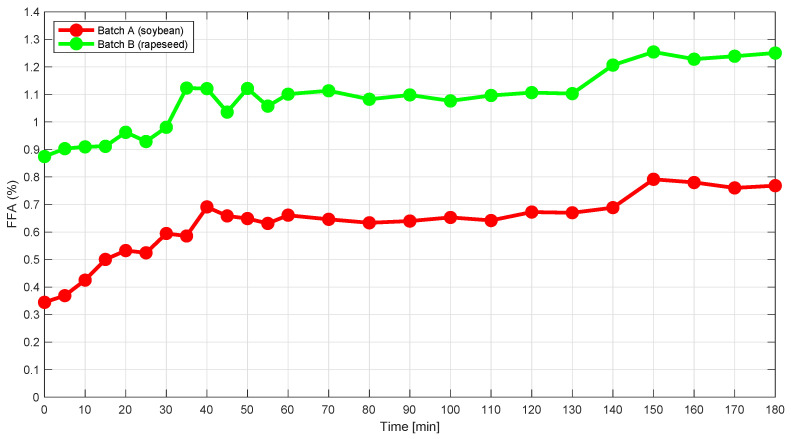
Graph showing the FFA content (titration) in percent (*w*/*w*) as a function of time for the two degumming reactions A) Soybean and B) Rapeseed. The estimated uncertainty of the FFA method is approximately 10%.

Figure 3 shows the FFA content as a function of time. Both batches have a plateau from around 60 to 140 min, after which only a slight increase in FFA is observed. The marked increase in FFA after approximately 130–150 min is speculated to be a result of a change in the physico-chemical and emulsifying properties when the phospholipids are gradually hydrolyzed to lyso-phospholipids. Phospholipids mainly form water-in-oil emulsions, whereas lyso-phospholipids form oil-in-water emulsions. The transition from a water-in-oil emulsion to an oil-in-water emulsion may explain the increase in FFA, as the phospholipids become more available for hydrolysis.

### 3.2. Spectral Features of the Vegetable Oils

**Figure 4:**
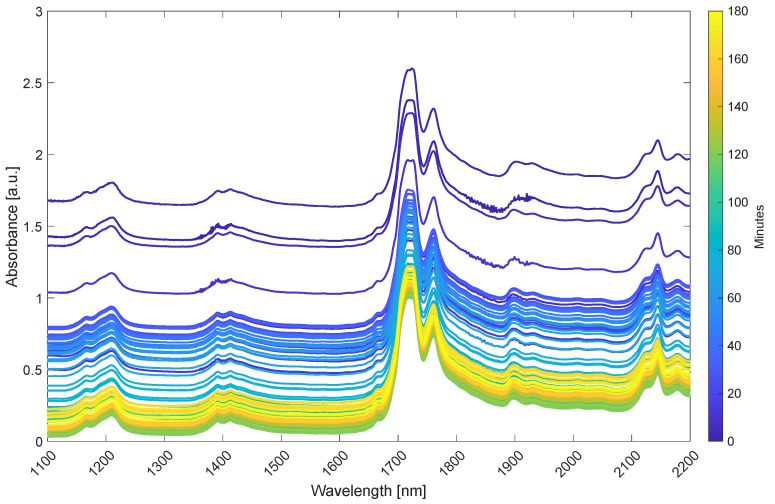
Single scan NIR spectra of batch A colored by time. Every 50th spectrum is plotted for clarity.

The near-infrared region is characteristic in containing the combination and overtones of the fundamental molecular vibrations absorbing in the infrared region. As NIRS is more sensitive to anharmonic vibrations, e.g., C-H, N-H and O-H vibrations, and contains repeated redundant information [29], it can typically be used for more robust calibrations. Figure 4 shows the single scan NIR spectra of all samples in batch A colored by time (only every 50th spectrum is plotted for clarity). Different regions of absorption can be observed in the spectrum, with different intensities corresponding to the oil and the products formed in the hydrolysis reaction. The main peaks between 1700 and 1800 nm correspond to the first overtone of C-H stretching vibrations and are characteristic for oils. The shape of the peaks in this region differs slightly between different oil types due to differences in the fatty acid composition [9]. The complex overlapped peaks between 1800 and 2200 nm correspond primarily to O-H combination bonds, showing the presence of O-H in glycerides and phospholipids, but also the moisture present (H_2_O). However, the region also contains peaks for O-H vibrations from P-OH stretching vibrations (1910 nm, 1st overtone) and CH + C=C combination bands from unsaturated fats (2150–2200 nm). At 1350–1500 nm, the combination bands from C-H deformation and C-H stretching vibrations are present together with the second overtone of O-H stretching vibrations around 1450 nm. At 1150–1250 nm, bands corresponding to the second overtone of C-H stretching vibrations are present. These are all typical for lipid/oil systems.

### 3.3. Exploration of the Degumming Process by PCA

The appearance of the PCA score plots is highly influenced by the choice of spectral preprocessing. Averaging with a process window width of 30 s, i.e., the minimum average measurement time investigated, shows a significant influence on both the appearance and explained variance as compared to no averaging (window width = 0). However, no significant difference in appearance and explained variance is observed when using a window width above 30 s (data not shown). As no quantitative tool for determining the optimal window width is available, a compromise between experimental noise and process dynamics must be made. The data suggest that a window width of 30–90 s provides an effective reduction of experimental noise without losing details of the process dynamics. This time range corresponds approximately to averaging 16–48 scans with the interferometer.

**Figure 5:**
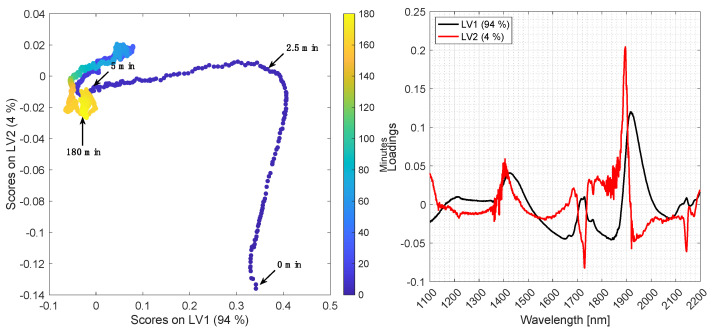
Left: PCA score plot of batch A using EMSC preprocessing and a measurement time window width of 60 s. Right: Loadings plot of LV1 and LV2.

**Figure 6:**
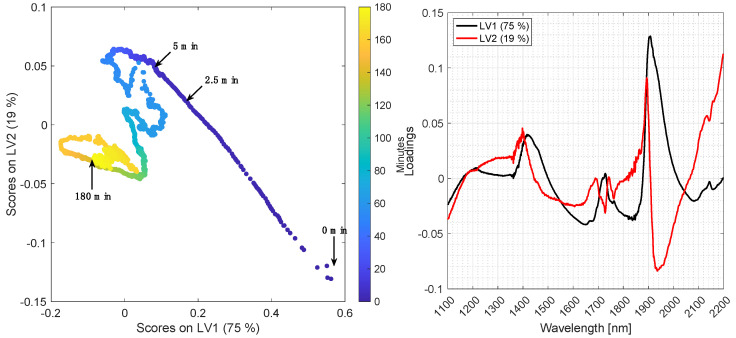
Left: PCA score plot of batch B using EMSC preprocessing and a measurement time window width of 60 s. Right: Loadings plot of LV1 and LV2.

Figure 5 and Figure 6 show the PCA score plot (LV1 vs. LV2) (left panel) and loading plot (right panel) for batch A and B, respectively. Data were averaged with a window width of 60 s and preprocessed by EMSC and mean centering. The scores are colored according to time. LV1 and LV2 explain 98% and 94% of the variation of the spectra recorded from batch A and B, respectively.

In batch A, a major transition takes place along LV2 in the first two minutes, and then changes to go along LV1, which is the most important based on explained variance. The major transition in batch B is moving diagonally along LV1 and LV2 within the first 5 min. During degumming of batch B, air bubbles were trapped in the light path for short periods of time, and for a longer period from approximately 40–50 min. These spectra of air were automatically removed as described in the Section 2.6 and is the reason for missing points in some parts of the trajectory in the score plot.

The loadings in Figure 5 and Figure 6 (right) reflect the overall variation of the enzymatic hydrolysis process. LV1 is predominantly influenced by the first overtone of O-H stretching vibrations (1400–1425 nm), together with the O-H combination tone (1900–1920 nm), which signifies that water is being consumed in the hydrolysis process, as the loadings are positive and the scores go from positive to negative. LV2 is mainly influenced by the first overtone of C-H stretching vibrations (1700–1800 nm) and the second overtone of C=O stretching vibrations (1900 nm) related to the carboxylic acid functional group present in free fatty acids. This corresponds to an increase in free fatty acid concentration by closure.

Pan et al. 2000 investigated the kinetics of degumming processes of sunflower oil by following the concentration of different phospholipids with HPLC. The oil was first degummed with 2.5% water at 40 °C for 55 min, resulting in a significant reduction of the total phospholipid content after 35 min. However, it was found that different phospholipids were hydrolyzed at different rates, due to different hydration rates [2,3]. For instance, the phosphatidylcholine (PC) content decreased by approximately 99% within 5 min, which agrees with the biggest transition in the PCA score plots in Figure 5 and Figure 6. In contrast, phosphatidylethanolamine (PE) was much slower to hydrolyze and not completely hydrolyzed even after 55 min of reaction [30]. Subsequently, the water degummed oil was degummed with 2.5% citric acid at 70 °C for 35 min, resulting in a significant reduction of the total phospholipid content after 15 min. Phosphatidylcholine (PC) and phosphatidic acid (PA) were present only in traces after 5 min, whereas phosphatidylethanolamine (PE) content was decreased to trace levels after 25 min.

The relative distribution of phospholipids in soybean oil (batch A) estimated by ^31^P-NMR is approximately 31.5% PC (phosphatidylcholine), 18.1% PI (phosphatidylinositol), 33.6% PE (phosphatidylethanolamine) and 16.8% PA (phosphatidic acid), and approximately 45.4% PC, 24.5% PI, 25.4% PE and 4.7% PA in rapeseed oil (batch B). PC has the highest relative rate of hydration (100%), followed by PI (44% of PC), PE (24% of PC) and PA (8.5% of PC) [3]. The higher content of PC in batch B may explain the faster transition visible in the PCA score plot, and additionally, the differences in hydration rates may explain the recurrent transition points from 5 to 180 min in the score plots.

### 3.4. Prediction of Free Fatty Acid Content

**Figure 7:**
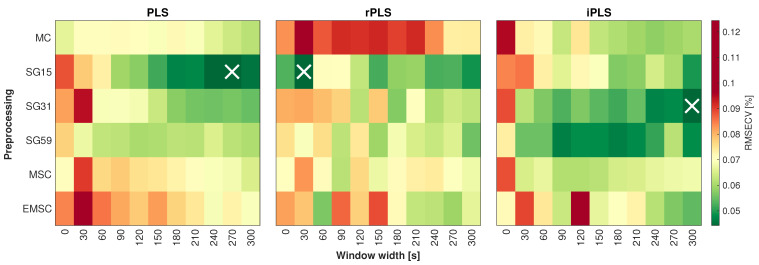
Response surface plot for PLS, rPLS and iPLS models (left, middle and right column). The preprocessing method is plotted against the averaging window width. The color scale corresponds to the RMSECV at the five latent variables (LVs) included. The optimal models, i.e., lowest RMSECV-value, are marked with a X.

Calibration models were established to investigate the effect of window width and preprocessing method on the regression performance. Figure 7 shows the PLS, rPLS and iPLS response surface plots for each combination of window width and preprocessing. The number of latent variables included is fixed to five for easier comparison of model performance. Table 2 shows an overview of the optimal calibration models with relevant model performance parameters.

The optimal model, including all spectral variables (i.e., no variable selection), had a RMSECV of 0.06% (*w*/*w*) FFA using five latent variables. Data were averaged using a window width of 270 s and preprocessed by Savitzky-Golay (15,2,2). Subsequently, two different methods of variable selection were tested for potential performance gains, namely recursive PLS (rPLS) and interval PLS (iPLS). The optimal model using rPLS was found to include eight wavelengths and had a RMSECV of 0.05% (*w*/*w*) FFA using five latent variables. The data were averaged using a window width of 30 s and preprocessed as second derivative spectra (Savitzky-Golay (15,2,2)). The optimal iPLS model included 300 spectral variables and had an RMSECV of 0.05% (*w*/*w*) FFA using five latent variables. Data were averaged using a window width of 300 s and preprocessed as second derivative spectra (Savitzky-Golay (31,2,2)).

**Figure 8:**
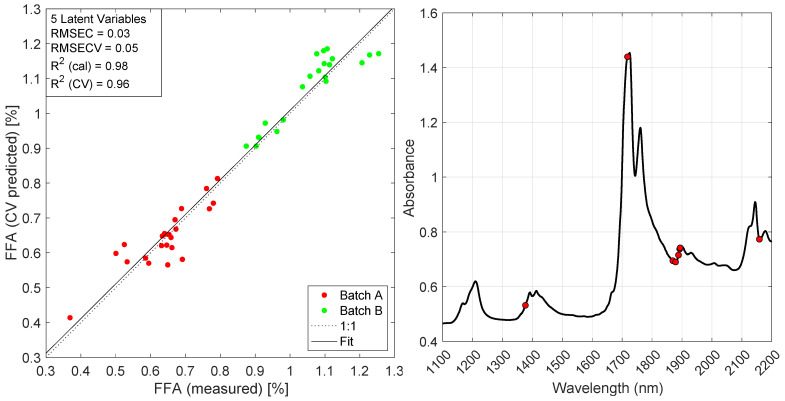
Left: Measured vs. predicted plot of optimal rPLS model (30 s averaging, SG15,2,2, 5 LVs; see Figure 7). Right: Average spectrum with the eight wavelengths chosen by the rPLS algorithm as indicated in red (1377, 1718, 1869, 1879, 1888, 1892, 1894 and 2159 nm).

The measured vs. predicted plot and the variables selected by rPLS are shown in Figure 8. The eight wavelengths selected by the rPLS algorithm are 1377, 1718, 1869, 1879, 1888, 1892, 1894 and 2159 nm.

**Figure 9:**
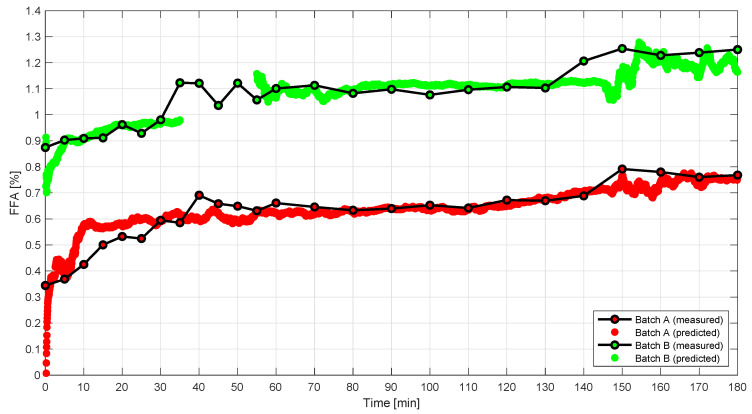
Predicted FFA content using the optimal calibration model (rPLS model with eight variables included and five LVs) on data averaged with a window width of 150 s. The predictions of batch A and B are colored in red and green, respectively. The reference values are plotted as black lines.

The optimal calibration model was used to predict the FFA content for every spectrum obtained for each batch. In that way, it was possible to obtain a prediction trajectory to compare with the reference values. The predictions are shown together with the reference values in Figure 9. The spectral data used for prediction were averaged using a window width of 180 s to reduce the influence of noise. In general, the predictions follow the measured FFA content reasonably well. However, deviations between predicted and measured FFA content are observed during the first 30 min of reaction in batch A. The gap in predictions in batch B from approximately 35 to 55 min is due to removal of non-informative spectra, as air bubbles were trapped in the sample cell.

Disagreement between predicted and measured FFA content is also observed at the end of the process, i.e., from 130 to 180 min of reaction in batch B. The predictions indicate that the change taking place is not detected by NIR in a timely fashion. This is likely due to increased level of noise in the spectra.

## 4. Discussion

The application of NIRS to monitor and predict constituents of vegetable oil has been studied in the literature, especially as related to the production of biodiesel. Rapid in-line and on-line NIRS methods have been evaluated as being successful for monitoring the transesterification reaction in the production of biodiesel from vegetable oil [10,12,14]. Bendini et al. [31] investigated the use of NIRS to predict fat content, moisture and free acidity (FA) in industrial olive oils. A total of 322 spectra were acquired in transmission mode and preprocessed as first derivative spectra (Savitzky-Golay 17,1,1) and baseline correction. Free acidity was predicted by PLS regression with an RMSECV of 0.03% (*w*/*w*) and an r^2^ of 0.986 in the range of 0.04–1.96% using nine latent variables in an optimistic leave-one-out cross-validation scenario. Rodrigues et al. [7] predicted the FFA content in refined soybean oil by NIRS using a PLS calibration model that included seven latent variables with an RMSECV of 0.88% (*w*/*w*) and an r^2^ of 0.99, using baseline correction and preprocessed as first derivative spectra (Savitzky-Golay 9,1,1). However, the range of measurements in the investigation covered 0–90% (*w*/*w*) FFA content, which differs significantly from the much smaller and narrower range investigated in this study (0.34–1.25% (*w*/*w*) FFA).

For the industrial degumming process, the FFA represent the most obvious and direct chemical components for the establishment of calibration models. In industrial production plants, FFA calibration models will also be straightforward to maintain and update, since the FFA determination by titration is normally available. The present study shows that calibration of NIR spectra to predict FFA content was indeed successful, although partially indirect in nature, since the consumption of water also play a significant role in the calibration models. It is not abnormal nor performance-deteriorating to build PLS regression models on indirect correlations, assuming that the indirect correlations rely on inherent biological or chemical relationships; this may be called the chemical cage of covariance [32].

During these experiments, spectral acquisition was carried out as fast as the instrument allowed (i.e., every 2–8 s) and subsequently averaged using different window widths to obtain a compromise between experimental noise and process dynamics, with temporal averaging necessary to reduce the experimental noise. In the present study, each spectrum in the window was weighed equally as a simple moving average (boxcar filter). Initially, two other weighted moving average functions were evaluated to investigate the effect of assigning higher weights to the most recent spectra. However, neither linear nor gaussian weighing functions provided better results (data not shown). The results obtained by PCA indicate that a window width of 30 s is sufficient and that a larger window width does not have a significant impact on the explained variance. This indicates that a normal procedure of averaging 16, 32 or 64 scans is suitable in most process monitoring situations. However, in processes with high dynamics, such as enzymatic processes, free-run spectroscopy can aid in the understanding and interpretation of these changes and potentially allow for monitoring rapid changes in the chemical system that would otherwise be hidden.

The calibration routine used in this study was fully automated, i.e. both outlier detection, determination of the number of latent variables and variable selection were done programmatically, with a minimum of or no user input. However, the detection of outliers is based on a 95% confidence interval to exclude extreme samples from the calibration model. As no reliable automatic method exists for the automatic selection of the optimal number of components, we did not automate this process but rather based it on manual selection. An alternative method for outlier detection based on the influence plot (Hotelling’s T^2^ vs. Q residuals) was investigated, but this procedure did not result in improved prediction models; however, it may have the potential for more robust model generation.

The main challenge in this calibration problem is that the reaction changes fast at the beginning of the process (the first 0–5 min) as is visualized in the PCA score plot in Figure 5 and Figure 6. Since reference samples were only collected every fifth minute during the reaction, it is difficult to establish PLS regression models including all samples, as the first reference samples in each batch show outlying behavior due to extreme reference values. This is also evident from Figure 9, which shows the predictions. A solution to this challenge would be to take out reference samples more frequently during the first 30 min of each batch, e.g., every second minute.

The relative standard deviation (RSD) of the FFA titration method is estimated to be in the range of 10–15%. As the RMSE summarizes both the error arising from the reference and the calibration model, the established prediction models may perform better than the reference method. To confirm this, the FFA content should be quantified using a more accurate method, e.g., GC-MS [33] or SFC [34].

Via optical fibers, NIR instrumentation can be installed at one or several places in the process line to enable real-time process monitoring and control. Application of PAT principles such as feed-forward and feed-back systems will enable the possibility of fully automatic process control, e.g., automatic enzyme dosage [16]. In addition, on-line monitoring of the process gives real-time insight into the process kinetics, as demonstrated in this study. NIRS facilitates rapid and constant monitoring of the process and enables establishment of control charts. Thus, immediate action can be taken, and the production can be halted/changed if the quality is not acceptable, i.e., if the degumming is insufficient. Moreover, if it is possible to monitor the concentration of the different phospholipids, it can be made clear which part of the hydrolysis reaction is not proceeding optimally. This information can be used to optimize the conditions during degumming to obtain a more effective process.

## 5. Conclusions

This study demonstrated that it is possible to monitor the rapid dynamics of enzymatic degumming reaction with free-run NIR spectroscopy. The effect of window width in process time-domain averaging and spectral preprocessing was investigated by PCA and PLS modelling. Models established on process-averaged data showed significantly better performance in comparison to traditional models based on unsmoothed (raw) data. Independently of the averaging window width, strong transition points were observed in the PCA score plots within the first 5 min of reaction. This signifies a very high rate of conversion at the onset of the enzymatic reaction, underlining the benefit of free-run NIR spectroscopy. A robust PLS calibration model was established, enabling determination of free fatty acid content during production, with an RMSECV of 0.05% (*w*/*w*) FFA using five latent variables and eight spectral variables.

## Figures and Tables

**Table 1:** Statistical data on reference FFA measurements. SD = standard deviation, RSD = relative standard deviation = (SD/mean)·100.

	Batch A	Batch B	Batch A + B
Origin	Soybean	Rapeseed	Soybean/Rapeseed
Number of samples	25	25	50
FFA: Mean ± SD (% *w*/*w*)	0.62 ± 0.12	1.08 ± 0.11	0.85 ± 0.26
FFA: Range (% *w*/*w*)	0.34–0.79	0.87–1.25	0.34–1.25
RSD (%)	19.4	10.2	30.3

**Table 2:** Overview of the optimal NIRS calibration models for FFA. # LVs: number of latent variables, RMSEC: Root Mean Square Error of Calibration, RMSECV: Root Mean Square Error of Cross Validation.

Model	Window Width	Preprocessing	# Samples	# Variables	# LVs	RMSEC (%)	r^2^ (CAL)	RMSECV (%)	r^2^ (CV)
PLS	270	SG15,2,2	44	1178	5	0.04	0.97	0.06	0.94
rPLS	30	SG15,2,2	44	8	5	0.03	0.98	0.05	0.96
iPLS	300	SG31,2,2	44	300	5	0.03	0.98	0.05	0.96

## Data Availability

Data is available on request.
